# A Comprehensive Analysis of the Alternative Splicing Co-Factor U2AF65B Gene Family Reveals Its Role in Stress Responses and Root Development

**DOI:** 10.3390/ijms26083901

**Published:** 2025-04-20

**Authors:** Xiangfeng Meng, Yongzhou Wang, Bei Tang, Jie Zhou, Yangfan Gu, Qingqiu Shen, Yaqun Zhou, Baohua Wang, Hui Fang, Yunying Cao

**Affiliations:** College of Life Sciences, Nantong University, Nantong 226019, China; mxf136463@163.com (X.M.); wangzizhouzi@163.com (Y.W.); 15212821529@163.com (B.T.); 2209110216@stmail.ntu.edu.cn (J.Z.); gyf289912@outlook.com (Y.G.); 19552625606@163.com (Q.S.); cd200041250@126.com (Y.Z.); bhwang@ntu.edu.cn (B.W.)

**Keywords:** U2AF65B, splicing, abiotic stress response, expression pattern, loss of function

## Abstract

U2AF65, a 65 kDa splicing co-factor, promotes spliceosome assembly. Although its role in alternative splicing (AS) is known, the function of U2AF65B (the large subunit of U2AF65) remains unclear. Therefore, we systematically identified and analyzed the U2AF65B gene family across 36 plant species, revealing 103 putative members with conserved structures and functions. Phylogenetic analysis divided the genes into two clades and five subgroups, indicating evolutionary divergence. Gene structure and conserved motif analyses showed that most *U2AF65B* genes have complex structures and shared similar motifs. Homology modeling and amino acid conservation analyses revealed significant conservation in U2AF65B amino acid sequences, particularly in Groups D and E. *Cis*-acting element analysis indicated that *U2AF65B* genes respond to various stimuli, supported by expression analysis under different stress conditions. Subcellular localization predictions indicated that U2AF65B proteins primarily localize in the nucleus and the cytoplasm. Alternative splicing (AS) profile analysis showed that the AS frequency likely varies between species. Functional analysis of the *AtU2AF65B* mutant in *Arabidopsis* revealed that AtU2AF65B function loss enhances root elongation and attenuates ABA-dependent germination suppression, indicating negatively regulated seedling growth and development. These findings provide insights into the evolutionary history, molecular mechanisms, and functional roles of the U2AF65B gene family in plants.

## 1. Introduction

The evolutionary transition of plants from aquatic algae to terrestrial angiosperms is characterized by revolutionary adaptive innovations. Dicotyledons (e.g., *Arabidopsis thaliana* and *Glycine max*) and monocotyledons (e.g., *Oryza sativa* and *Zea mays*) have colonized diverse terrestrial niches via seed-based reproduction and specialized floral structures [1]. Ferns (such as *Selaginella moellendorffii*) developed vascular systems for efficient water transport [2], while algae (such as *Chlamydomonas reinhardtii* and *Volvox carteri*) retain ancestral aquatic features, providing critical insights into plant terrestrialization [3]. Within this evolutionary framework, innovations in precursor messenger RNA (pre-mRNA) splicing mechanisms have been identified as pivotal adaptions for plant survival in dynamic terrestrial environments.

Pre-mRNA splicing is a critical step in eukaryotic gene expression that involves removing introns and connecting exons to generate mature mRNA [4,5]. This process is mediated by the spliceosome, a complex for eukaryotic cell function [6,7,8], including the major spliceosome (U1, U2, U4, U5, and U6 small nuclear ribonucleoprotein (snRNPs)) and the minor spliceosome (U5, U11, U12, U4atac, and U6atac) [9,10,11,12]. The interaction between the U1 snRNP and the 5′ splice site (5′ SS) influences the binding of U2 small nuclear RNA auxiliary factor 65 (U2AF65) to the downstream 3′ splice site (3′ SS) [13]. The pyrimidine tract (Py tract), located between the AG dinucleotides at the 3′ SS and the branch point, is rich in pyrimidine bases (cytosine and uracil) and guides spliceosome assembly at the 3′ SS [14]. The U2 snRNP particle auxiliary factor (U2AF) is crucial for pre-mRNA splicing [15]. In a human genome study, researchers observed that the high-affinity binding sites of U2AF65 strongly inhibit exons, suggesting that splicing occurs against a backdrop of inhibition [16].

The human essential splicing factor U2AF consists of a 35 kDa subunit (U2AF35) and a 65 kDa subunit (U2AF65), forming heterodimers [17]. U2AF65 contains three RNA recognition motifs (RRMs) at its C-terminus, and an N-terminal arginine/serine-rich (RS) domain [18]. The two central RNA recognition motifs (RRM1 and RRM2) in U2AF65 recognize the common polypyrimidine tract (PPT) sequence in pre-mRNA [19], while an atypical C-terminal RRM domain forms the U2AF Homology Motif (UHM) [20]. Its core features a βαββαβ topology, with an α/β sandwich arrangement comprising four antiparallel β-strands and two stacked α-helices [21]. Functioning as an atypical RRM domain, the UHM mediates crucial protein–protein interactions and regulates alternative pre-mRNA splicing and other processes. The UHM ligand motif (ULM) peptide sequence is crucial for recognizing the UHM domain during early spliceosome assembly [22]. During spliceosome assembly, U1 snRNP initially binds to the 5′ SS [23], U2AF35 specifically recognizes the AG dinucleotide at the 3′ SS [24], and U2AF65 binds to the adjacent Py tract via its RRM1 and RRM2. Additionally, U2AF65 enhances binding by interacting with the N-terminal ULM domain of splicing factor 1 (SF1), the C-terminal UHM domain, and the branch point sequence (BPS) through its N-terminal RS domain [25,26]. The RS domain facilitates various RNA–RNA interactions, including base pairing between the BPS and U2 snRNA [27]. U2 snRNPs then replace SF1, bind to the BPS, and recruit U5/U4/U6 snRNPs to complete spliceosome assembly. Spliceosomes remove introns and ligate exons through two consecutive transesterification reactions [28].

U2AF65 promotes alternative exon exclusion through spliceosome-inhibitory effects and facilitates pre-mRNA assembly by interacting with the PPT. These effects can be observed in the pre-mRNAs of survival motor neurons associated with spinal muscular atrophy and the apoptosis factor (Fas) [29]. Additionally, U2AF65 inhibits the splicing of late adenovirus and β-globin precursor mRNAs, leading to alternative exon skipping [29]. Furthermore, studies have shown that a 56 kDa U2AF-associated protein (UAP56), cloned from *Saccharomyces cerevisiae*, plays a crucial role in splicing by recruiting pre-mRNA and facilitating U2 snRNP branch point interaction [30]. However, studies on the role of U2AF65 in plants are limited. Two *U2AF65* genes (*U2AF65A* and *U2AF65B*) have been identified in *Arabidopsis* and *Oryza sativa* [31,32], suggesting that various U2AF subtypes are widespread in plants [32]. The requirements for intron recognition during pre-mRNA splicing in plants differ from those in vertebrates and yeast. U2AF65 binds to the PPT and is displaced to RNA-binding sites. It interacts with SF1, facilitating the recruitment of U2 snRNP and stabilizing the interaction between U2 snRNP and the intron branch point in spliceosome complex A [33]. Plant introns lack both the conserved branch points and the proximal polypyrimidine tract (PPT) characteristic of vertebrate introns, while their 3′ splice sites resemble those of yeast introns [34]. Plants, like animals and yeast, have introns rich in uridine (U) residues, a necessary characteristic for splicing. *U2AF65* in tobacco interacts with RNA segments containing introns and exhibits affinity for poly (U), poly (C), and poly (G). However, neither the BPS nor the 3′ SS region significantly affects intron recognition by *NpU2AF65* [35]. *Arabidopsis U2AF65* (*AtU2AF65*) regulates flowering time and pollen tube growth, while the *AtU2AF65A* mutant exhibits a delayed flowering phenotype [36] and is involved in ABA-mediated flowering transitions [37].

Recent studies highlight the role of pre-mRNA splicing factors in plant adaptation to abiotic stresses. Ambient temperature fluctuations trigger AS events in splicing-related genes [38]. For instance, the splicing regulatory factor *RCF1* ensures accurate pre-mRNA splicing. Under low-temperature conditions, the *rcf1-1* mutant aberrantly splices core cold-responsive genes, such as *CBF* (C-repeat binding factor), compromising cold signaling pathway integrity in *Arabidopsis* [39]. The spliceosome component *SKIP* directly participates in processing the mRNA transcripts of stress-related genes. In *Arabidopsis,* the *skip-1* mutant shows increased sensitivity to salt and osmotic stress due to impaired AS in salt-tolerance genes [40]. In *Populus*, the splicing factor *PtU1-70K* improves plant detoxification capacity under heavy metal stress by modulating the AS of *PtHSP70*, thereby generating stress-specific heat shock protein isoforms [41]. Similarly, the rice splicing factor *OsSCR106* ensures splicing accuracy under salt, ABA, and cold stress conditions, maintaining developmental homeostasis. The *osscr106* mutant exhibits multi-stress hypersensitivity, underscoring its role in stress adaptation [42]. Collectively, these findings demonstrate how splicing factors dynamically regulate AS to facilitate plant adaptation to abiotic stresses. Despite these insights, the specific functions of U2AF65 isoforms (U2AF65A and U2AF65B) in plants remain unclear.

Herein, we conducted a comprehensive analysis of *U2AF65B* in various species, including investigating its evolutionary relationships, gene and protein structures, and expression patterns under different stresses and performing an AS analysis. The results of this study will contribute to a deeper understanding of the regulatory roles of U2AF65B genes in response to stress.

## 2. Results

### 2.1. Genome-Wide Identification and Phylogenetic Analysis of the Plant U2AF65B Gene Family

**Figure 1:**
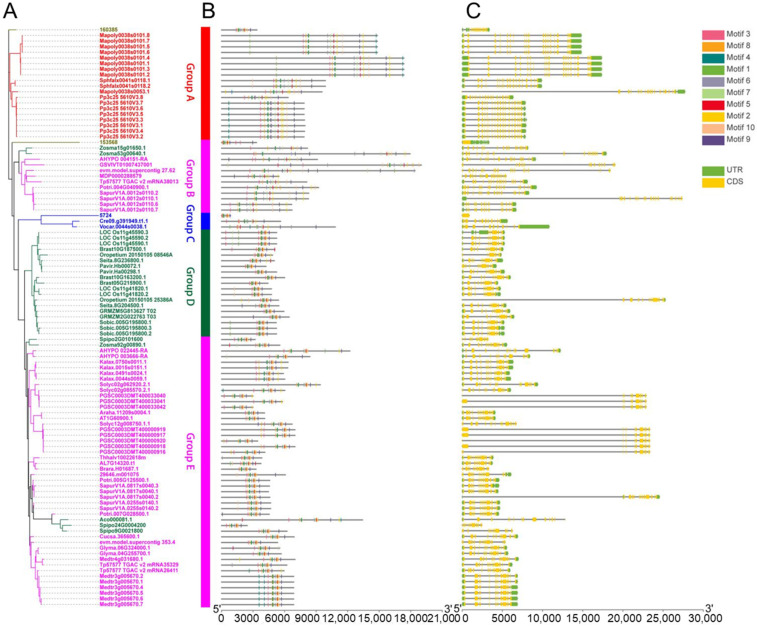
Genomic structures and conserved motifs of plant *U2AF65B* genes among various species. (**A**) Phylogenetic tree of U2AF65B proteins. The vertical line in the phylogenetic tree marks a branch break. Colors: red for bryophytes, olive green for pteridophyta (Group A), pink for dicotyledons (Groups B and E), blue for algae (Group C), and green for monocotyledons (Group D). (**B**) Conserved motifs identified via MEME analysis. (**C**) Gene structure visualization. Green bars represent UTRs of genes, yellow bars represent the exons, and horizontal lines connecting the bars represent the introns.

To identify *U2AF65B* genes across species, we conducted a BLAST search from Phytozome v12.1.6 (https://phytozome.jgi.doe.gov) (accessed on 21 September 2022), yielding 103 putative U2AF65B sequences from 36 species. Detailed information for each sequence is provided in Table S1. Specifically, the sequences comprised 10 monocotyledons (green), 19 dicotyledons (pink), 3 algae (blue), 3 bryophytes (red), and 1 pteridophyte (olive green) (Figure 1A). The phylogenetic tree was divided into two clades: Clade I (containing Group A) and Clade II (containing Groups B, C, D, and E) (Figure S1). These clades were further divided into five groups—Group A (mainly bryophytes), Groups B and E (dicotyledons), Group C (algae), and Group D (monocotyledons)—indicating the evolutionary divergence of *U2AF65B* genes across species. Among 36 species, *U2AF65B* gene numbers varied: 13 species had one gene, 8 species had two, 8 had three, *Kalanchoe laxiflora* had four, *Oryza. sativa* had five, *Medicago truncatula* had seven, 2 species (*Physcomitrella. patens* and *Solanum tuberosum*) contained eight, and 2 (*Marchantia polymorpha* and *Salix purpurea*) had nine.

To explore evolutionary relationships, we built a phylogenetic tree of 103 U2AF65B proteins (Figure 1A). In the phylogenetic tree, Group A (red) is the most basal lineage; Group D (green) includes monocotyledons; and Group E (pink) primarily includes dicotyledons. Group C showed close affinity to Groups B and D, indicating evolutionary connections between algae, monocotyledons, and dicotyledons. Group A (red), dominated by bryophytes, is more distantly related to the others. Therefore, bryophytes and pteridophytes are distant from algae, monocotyledons, and dicotyledons. Sequences from the same species often cluster. Group C has only pteridophytes, and Group D contains only monocotyledons. However, Group A includes bryophytes and a subclass of pteridophytes (*S. moellendorffii*). Group E also contains three monocotyledons (*Ananas comosus*, *Spirodela polyrhiza*, and *Zostera marina*), and Group B (dicotyledons) included one pteridophyte (*S. moellendorffii*) and one monocotyledon (*Z. marina*). These observations likely reflect the evolutionary dynamics of these plants.

### 2.2. Comparative Analysis of Gene Structure and Conserved Motifs in the U2AF65B Gene Family

TBtools was used to analyze the gene structure and conserved motifs of the U2AF65B gene family in selected plants to clarify their evolutionary history and motif functions [43]. Conserved motifs, critical for protein function, were relatively uniform across 64% (66/103) of the genes. Additionally, *U2AF65B* genes in the same evolutionary branch shared similar conserved motifs (Figure 1B and Figure S2), and gene structures within subfamilies were largely conserved (Figure 1C), implying shared functions. The majority of *U2AF65B* genes had more than 10 exons. Notably, 17 genes lacked UTR regions (primarily in *S. tuberosum*), and 1 gene (*Ostreococcus lucimarinus*) lacked introns. These results align with the previously conserved gene structure of the U2AF65B family, suggesting that it preserves key functional characteristics and may regulate protein structures and functions.

### 2.3. Homology Modeling and Amino Acid Conservation Estimation

**Figure 2:**
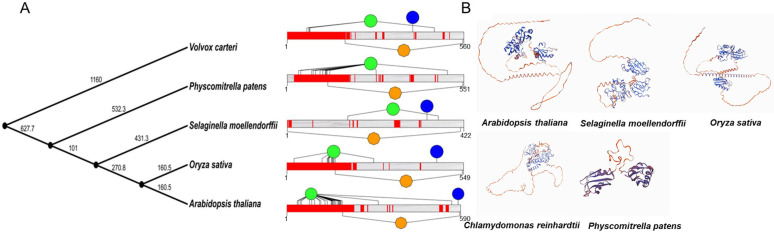
Structural analysis of U2AF65B proteins. (**A**) RRM domain (orange), disordered regions (red), Ser phosphorylation sites (green), and Tyr phosphorylation sites (blue) in U2AF65B proteins. Left: evolutionary tree with geographic time; right: protein sequence lengths. (**B**) A 3D model of U2AF65B proteins (using *Arabidopsis*, *Selaginella moellendorffii*, *Oryza sativa*, *Chlamydomonas reinhardtii*, and *Physcomitrella patens*). Model colored based on QMEAN quality scores (darker blue = higher sequence identity).

We performed multiple sequence alignments of U2AF65B proteins using the NCBI MSA Viewer and COBALT based on conserved domains and local sequence similarity (Figure S3). Using a frequency difference method, we analyzed the amino acid distribution and quantified the conservation level. The data revealed the significant conservation of U2AF65B amino acid sequences, especially in Groups D and E. All five species contain the RRM domain (Figure 2A). Notably, all U2AF65B proteins except those in *A. thaliana* exhibited disordered structures. Additionally, we used the neural network system NetPhoS v3.1 to predict phosphorylation sites in proteins from selected species (*A. thaliana*, *Oryza. sativa*, *P. patens*, *S. moellendorffii*, and *Volvox carteri*). The results revealed that U2AF65B across all species exhibited serine enrichment and tyrosine depletion, suggesting that its phosphorylation potential may be limited by the accessibility of specific amino acid residues (Figure 2A and Figure S4).

Next, we constructed 3D models of U2AF65B proteins from five selected species using homology modeling on the SWISS-MODEL platform (Figure 2B). The 3D model exhibited conserved RRM structures consistent with the results of the amino acid sequence alignments. Additionally, proteins from *P. patens* exhibited significant differences in their 3D structures, likely due to evolutionary divergence. In contrast, proteins in other species exhibited similar 3D structures.

### 2.4. Analysis of Cis-Acting Elements in U2AF65B Promoter Sequences

The 1.5 kb promoter sequence of *U2AF65B* genes was retrieved from the Phytozome database, and the *cis*-acting elements were analyzed using PlantCARE. Of the 12,944 *cis*-acting elements identified in 103 genes, 9222 (71.2%) remained after removing 1013 blank and 1119 unnamed elements. TBtools was used to construct a distribution map of the *cis*-acting elements and to integrate it with the phylogenetic map (Figure S5). The results showed that *cis*-acting elements were broadly distributed across most genes, with the AT-TATA-box being the most abundant. This element is a key transcriptional component common in plants (Figure S5). However, certain species exhibited variations in *cis*-acting elements, such as *Glycine Max*, *Marchantia*, *M. truncatula*, *P. patens*, and *S. purpurea*.

**Figure 3:**
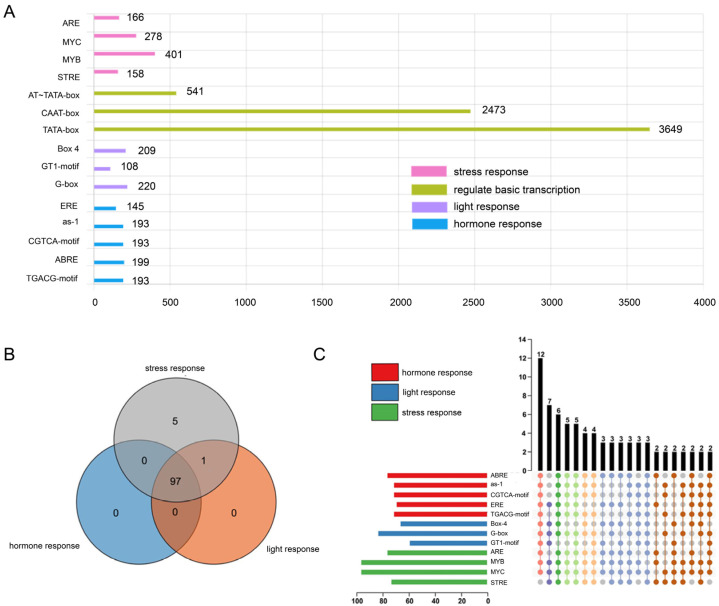
Analysis of *cis*-acting elements in *U2AF65B* promoters. (**A**) Function and number statistics of *cis*-acting elements. (**B**) Overall enrichment of *cis*-acting elements in response to stress, hormones, and light. (**C**) Specific enrichment of stress-, hormone- and light-responsive *cis*-acting elements, different colors represent the number of set intersections.

The 9222 *cis*-acting elements were categorized into 15 types (Figure 3A) and further divided into four categories: hormone response (923 elements), light response (537 elements), basic transcription regulation (6663 elements), and stress response (1003 elements). One element (96 AAGAA-motif) was not clearly defined. The hormone-responsive elements included 145 ethylene response elements (EREs), 193 as-1 elements, 193 CGTCA-motifs (involved in the methyl jasmonate (MeJA) response), 193 TGACG-motifs (involved in the MeJA response), and 199 ABRE elements (involved in the abscisic acid response). Notably, the as-1 elements and the CGTCA and TGACG motifs were equally abundant and co-occurred, suggesting potentially similar functions. The light response elements included 209 Box4, 220 G-box, and 108 GT1 motifs. The basic regulatory elements included 3649 TATA-boxes (transcription start sites), 2473 CAAT-boxes (promoter and enhancer regulatory elements), and 541 AT-TATA-boxes. The stress response elements comprised 166 AREs (anoxia responses), 401 MYBs, 278 MYCs, and 158 STREs (osmotic stress responses). Among these elements, transcription-regulating elements (especially TATA and CAAT boxes) were most frequent, highlighting their key roles in plant transcriptional regulation. The abundance of hormone-responsive elements suggests that these plants respond to multiple hormones, thereby enhancing gene expression.

Furthermore, a stimulation enrichment analysis of the *cis*-acting element in *U2AF65B* promoters was conducted using TBtools (Figure 3B). All 103 *U2AF65B* genes responded to stress, 98 responded to light, and 97 responded to hormones. Notably, 97 genes (94%) responded to all three stimuli simultaneously, whereas only 5 genes from four plants (*Brachypodium stacei*, *S. tuberosum*, *V. vinifera*, and *Z. mays*) responded solely to stress. A more detailed analysis showed that only 1 gene contained all elements, 58 contained 9–11 elements, 42 contained 5–8 elements, and 2 contained 3–4 elements (Figure 3C). Overall, *U2AF65B* responded to multiple stimuli, forming a complex response system. Most sequences shared similar stress response mechanisms, while a few may have unique regulatory mechanisms or exist in different biological contexts.

### 2.5. Expression and Subcellular Localization of Plant U2AF65B Genes

**Figure 4:**
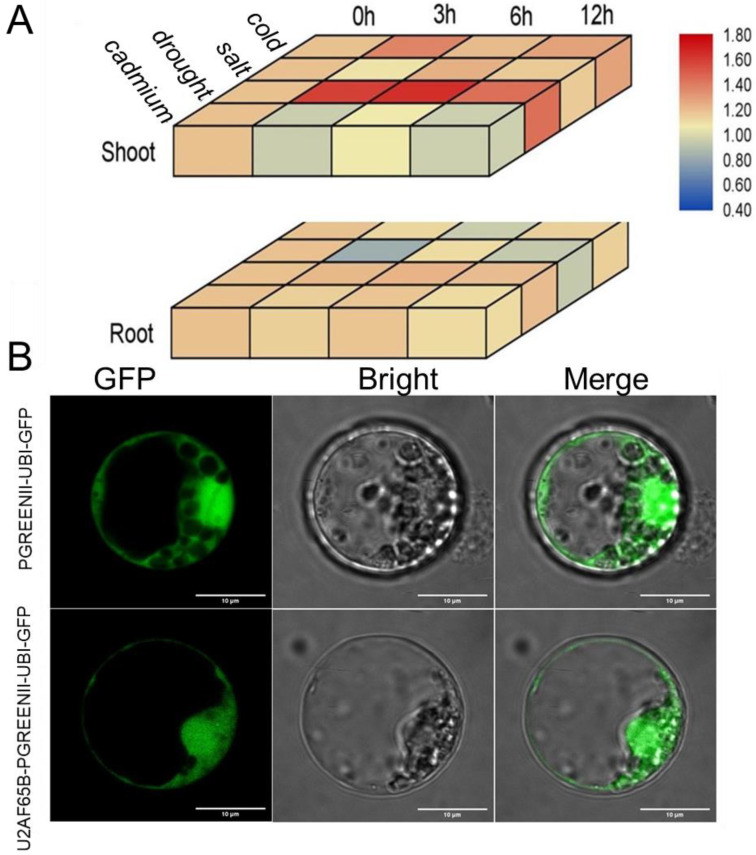
Expression and subcellular localization analysis of the *OsU2AF65B* gene in *O. sativa*. (**A**) Heatmap of *OsU2AF65B* gene expression (*LOC_Os11g45590.2*) in shoots and roots (log_2_-transformed data). Experiments were repeated at least three times. (**B**) Subcellular localization of OsU2AF65B. Rice protoplasts were transfected with OsU2AF65B-PGREENII-UBI-GFP and PGREENII-UBI-GFP. Green fluorescence represents the expression site of the fusion protein. Scale bar = 10 μm.

The presence of numerous stress response elements in the promoter region of *U2AF65B* genes suggests their potential role in plant stress responses. To test this hypothesis, we applied four stress treatments to *O. sativa*: cold, salinity, drought, and cadmium (Cd^2+^) (Figure 4A). Under cold stress, *U2AF65B* expression was higher in shoots than in roots, peaking at 3 h in shoots and reaching its lowest level at 6 h in roots (Figure 4A). Under salt treatment, *U2AF65B* exhibited significantly higher expression in shoots than in roots, while its expression in roots was down-regulated, reaching its minimum level at 3 h post-treatment. Under drought conditions, *U2AF65B* gene expression in shoots was upregulated from 3 to 12 h after treatments, while no significant change was observed in roots. Under the cadmium (Cd^2+^) treatment, *U2AF65B* expression in shoots at 3 and 12 h was significantly lower than in the control. Notably, both drought and Cd^2+^ treatments significantly altered *U2AF65B* expression in shoots. These results indicated that *U2AF65B* expression patterns in shoots and roots were inconsistent over a short period (12 h) under the four stress treatments, with more pronounced changes in shoots than in roots. Notably, shoots exhibit greater abiotic stress sensitivity than roots, implying that *U2AF65B* predominantly orchestrates plant stress adaptation through shoot-specific regulatory networks.

To clarify the function of U2AF65B, we predicted its subcellular localization in various plants (Figure S6). Approximately 80% of these proteins were primarily expressed in the nucleus, followed by the cytoplasm. Additionally, we selected the model plant rice to verify the gene expression site using protoplasts. An OsU2AF65B-PGREENII-UBI-GFP construct was generated using PGREENII-UBI-GFP as a control (Figure 4B). The results showed that the green fluorescence of the OsU2AF65B-PGREENII-UBI-GFP fusion protein was localized in the nucleus and the cytoplasm, confirming that OsU2AF65B functions in these locations.

### 2.6. Analysis of the Co-Expression Network of the OsU2AF65B Gene Under Drought Stress

**Figure 5:**
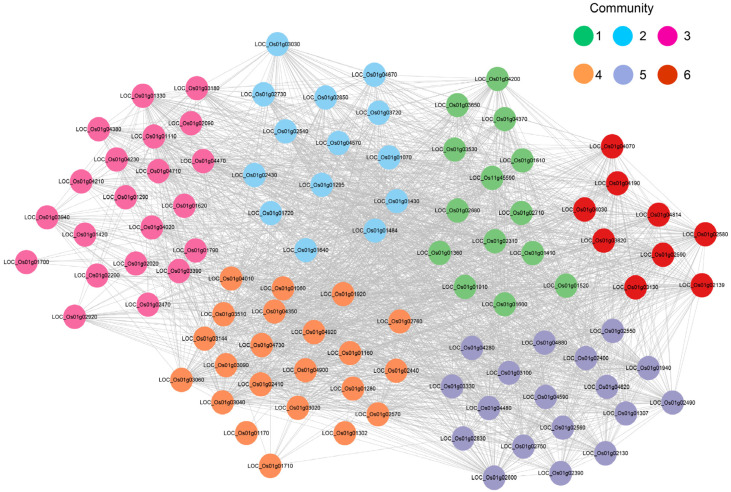
Co-expression network of the *OsU2AF65B* gene under drought stress. Different colors represent distinct communities.

Given the significant upregulation of the *OsU2AF65B* gene under drought conditions, we investigated its regulatory role using transcriptomic data from rice shoots under drought stress [44]. We identified 98 genes that interact with *OsU2AF65B* from 1119 DEGs through a co-expression network analysis (Figure 5). These genes can be grouped into six functional communities. Among them, *OsU2AF65B* and another 13 genes are in the same community. These genes include those related to transposon proteins, PTR2 peptide transporter proteins, immune response genes, and metabolic enzymes. These co-expression patterns indicate that *OsU2AF65B* may act as a regulatory hub governing genes linked to transposon-associated proteins, peptide transporters, and antioxidant enzymes to orchestrate drought stress adaptation mechanisms.

### 2.7. Analysis of AS Profile and Splicing Isoforms

To evaluate the evolutionary conservation and divergence of AS across plant lineages, we performed a comparative analysis using representative species from the major clades: four monocotyledons (*A. comosus*, *Oryza. sativa*, *Sorghum bicolor*, and *Z. mays*), three dicotyledons (*A. thaliana*, *G. max*, and *S. tuberosum*), three bryophytes (*Marchantia polymorpha*, *P. patens*, and *Sphagnum fallax*), and three algae (*C. reinhardtii*, *O. lucimarinus*, and *V. carteri*). The AS status of *U2AF65B* genes was assessed by comparing transcript structures retrieved from the Phytozome database (Figure S7). Among the selected plant species, AS exhibited the following distribution patterns. AS events were observed in three monocotyledons (*O. sativa*, *S. bicolor*, and *Z. mays*), two dicotyledons (*A. thaliana* and *G. max*), and all three bryophyte species (*M. polymorpha*, *P. patens*, and *S. fallax*). However, among algae, AS was only identified in *C. reinhardtii*. These results indicated that AS in *U2AF65B* genes occurred less frequently in algae. The frequent occurrence of AS in monocotyledons, dicotyledons, and bryophytes implied that these plants may use it to regulate gene expression and function, thereby adapting to different environments and physiological needs. Further analysis showed that *A. thaliana* had two *U2AF65B* genes, one of which exhibited AS characteristics, suggesting that even within the same species, different *U2AF65B* genes may have distinct splicing patterns and functions. These findings highlight the importance of AS as a regulatory mechanism in plant genomes, potentially playing a critical role in plant development and environmental adaptation.

### 2.8. Loss of AtU2AF65B Function Enhances Root Elongation and Attenuates ABA-Dependent Germination Suppression

**Figure 6:**
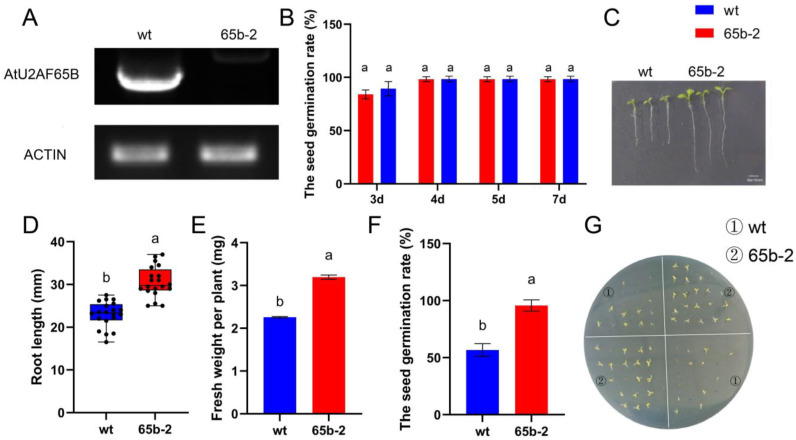
Phenotypic analysis of *AtU2AF65B* mutant in *Arabidopsis*. (**A**) RT-PCR identification of the *AtU2AF65B* mutant. (**B**) Germination rate in 1/2 MS medium (days 3–7, 50 seeds/replicate, 3 repeats). (**C**) Root length phenotype in 1/2 MS medium on day 7 (bar = 5 mm). (**D**) Root length in 1/2 MS culture medium (day 7, 20 seeds per replicate, three repeats). (**E**) Fresh weight of 20 *Arabidopsis* vertically grown plants over 7 days. (**F**) Germination rate in 1/2 MS + 1 μmol L^−1^ ABA for 13 days (40 seeds per replicate). (**G**) Germination phenotype in 1/2 MS + 1 μmol L^−1^ ABA for 13 days. Data in (**B**,**D**,**E**,**G**) are presented as the means ± SD of three independent experiments. Significant differences (*65b-2* vs. WT) are marked with lowercase letters based on independent-sample *t*-tests.

To further investigate the biological functions of *U2AF65B*, we conducted a comparative analysis of its protein sequences in rice and *Arabidopsis*. The results revealed high sequence conservation between rice and *Arabidopsis* U2AF65B, both containing the typical RRM domain (Figure S8). Based on this conserved feature, we selected an *Arabidopsis U2AF65B* mutant (*65b-2*) for phenotypic characterization. Detailed information on the T-DNA insertion site in this mutant is provided in Figure S9. RNA was extracted from 4-week-old *Arabidopsis* wild-type and mutant plants, and RT-PCR analysis did not detect any transcription products of *AtU2AF65B* in the *65b-2* mutant (Figure 6A). The germination rates of the mutant and wild-type plants grown in 1/2 MS culture medium were measured over several days, with no significant differences observed (Figure 6B). However, the mutant plants exhibited significantly greater root length (Figure 6C,D) and higher fresh weight per plant (Figure 6E) than the wild-type plants, suggesting that *AtU2AF65B* negatively regulates the growth and development of seedlings, particularly root length. After ABA treatment, the germination rate of the wild-type plants was significantly affected, differing from that of the mutant plants (Figure 6F,G). These results indicated that the *65b-2* mutation alleviated the inhibitory effect of ABA on seed germination. Therefore, *AtU2AF65B* may be a negative regulator in the ABA pathway during seed germination in *Arabidopsis*.

## 3. Discussion

### 3.1. The U2AF65B Genes Exhibited a Conserved Structure and Function

Gene family members often maintain conserved structures and sequences throughout evolution, performing essential biological functions crucial for organismal development and survival. In the present study, 103 *U2AF65B* members from 36 plant species (ranging from algae to angiosperms) were analyzed, with most genes from the same plant type clustering together in the phylogenetic tree. This finding can be linked to evolutionary relationships and structure conservation, suggesting that similar structures across species may perform essential biological functions. Therefore, the members of the same gene family likely have a low and relatively conserved structure [45]. Additionally, splicing variation is biologically significant, as it regulates gene expression. Specifically, splicing variations can alter protein structure and function, thereby regulating cellular physiological processes. For example, spliceosomes may vary in subcellular localizations, interaction partners, catalytic activities, or affinities, thus playing diverse roles in cell signaling, metabolism, and gene expression regulation [46,47]. AS is a key mechanism for generating functionally diverse proteins and regulating gene expression, indicating that *U2AF65B* expression is not highly variable. Although the splicing events were analyzed across species, accurate conclusions were limited by the small sample size. Previous studies have demonstrated that AtU2AF65B shuttles between the nucleus and the cytoplasm to fulfill its functions, consuming ATP during this process [35]. OsU2AF65B is localized to both the nucleus and the cytoplasm. This shuttling likely facilitates information or material exchange between these compartments [48]. It also allows OsU2AF65B to participate in nuclear processes like transcription and splicing while delivering information or substances to the cytoplasm, thereby affecting overall cell function.

As a vital auxiliary factor in plants, *U2AF65B* is essential for basic development and stress response in *Arabidopsis*. Previous studies indicate that *AtU2AF65B* regulates flowering by controlling ABA-related gene splicing [49] and is involved in light-induced seed germination in *Arabidopsis* [50]. The phenotypic analysis in this study showed that the U2AF65B loss-of-function mutant *65b-2* exhibited significant developmental remodeling, as evidenced by increased root elongation and enhanced fresh weight accumulation. These findings imply that *AtU2AF65B* may function by integrating auxin gradient formation or cell-elongation-related pathways, contributing to organ morphogenesis and resource allocation strategies [51]. Additionally, under exogenous ABA treatment, the germination rate of *65b-2* mutant seeds was significantly higher, suggesting that *AtU2AF65B* may act as a negative regulator in the ABA signaling pathway. Compared with the wild type, the *65b-2* mutant exhibited a significantly reduced sensitivity to ABA (Figure 6), confirming that U2AF65B finely regulates seed germination by inhibiting ABA signal transduction. This finding not only broadens our understanding of *AtU2AF65B*’s role in light-responsive germination [51] and the ABA pathway’s control of seed germination but also offers new insights into its function in ABA signaling.

### 3.2. Regulatory Roles of U2AF65B Genes in Plant Stress Responses

Promoter analysis showed that *U2AF65B* genes responded to stress, hormones, and light. The stress response elements included AREs, MYB, MYC, and STRE. AREs are *cis*-acting regulatory elements that induce hypoxia responses and regulate gene expression to help plants survive in low-oxygen environments. MYB and MYC are widely involved in plant growth, development, and responses to hormones and abiotic stressors, such as drought and salinity [52]. This suggests that *U2AF65B* genes primarily function via the MYB and MYC in these response processes. STRE is involved in responses to heat, pH, osmotic stress, and oxidative stress. While molecular mechanisms may vary between species, they often respond to stress by regulating *U2AF65B* expression [53]. Hormone response elements include the estrogen response element (ERE), as-1, the CGTCA motif, the TGACG motif, and the abscisic acid response element (ABRE). In higher plants, the as-1 element of the cauliflower mosaic virus 35S promoter mediates salicylic-acid- and auxin-induced transcription [54]. The CGTCA and TGACG motifs are *cis*-regulatory elements involved in the MeJA response. ERE is an enhancer in estrogen-responsive gene promoters [55], whereas ABRE functions in the ABA response. ABA, a key stress-signaling hormone, combats abiotic stress by regulating stomatal behavior [56]. This suggests that *U2AF65B* genes play a crucial regulatory role in plant flowering, development, and fruiting processes by responding to environmental changes via *cis*-acting elements. Furthermore, in this study, *U2AF65B* responded to various stimuli, including low temperature, salt stress, drought, and cadmium stress, showing notable sensitivity to salt, drought, and cold. Under these conditions, *U2AF65B* expression in shoots and roots exhibited distinct patterns in response to different stresses. *U2AF65B* expression in rice shoots and roots differed significantly in response to various stressors. Under cold and salt stresses, *U2AF65B* expression was higher in shoots than in roots, consistent with the expression pattern of *U2AF65A* [32]. Previous studies demonstrate that the splicing factor U2AF physically interacts with U1-70k [57]. U2AF65B may regulate the alternative splicing of the autophagy-related gene *PtoATG2* by interacting with PtoU1-70k and other serine/arginine-rich (SR) proteins. This interaction promotes the drought-resistant splice isoform *PtoATG2b* and triggers ROS overaccumulation, ultimately impairing stomatal aperture and drought tolerance [58]. Beyond its canonical role in per-mRNA splicing, *U2AF65B* integrates post-transcriptional and transcriptional stress response through epigenetic mechanisms, such as DNA methylation. Specifically, *U2AF65B* modulates cytosine methylation at the Methylation-Enabled Motif Site (MEMS) in the *ROS1* promoter, regulating ROS1-mediated DNA demethylation. This dynamic epigenetic modification fine-tunes stress-responsive gene expression during salt stress [59]. Co-expression network analysis revealed that *OsU2AF65B* clusters with transposon-related proteins and the PTR2 peptide transporter, implicating its role in suppressing cadmium uptake via root cells and enhancing vacuolar sequestration through the splicing regulation of heavy metal transporter genes [60]. Additionally, U2AF65B maintains genomic stability by suppressing cadmium-induced aberrant transposon jumping, thereby mitigating oxidative-stress-induced mutagenic risks [61].

By integrating the significantly upregulated expression of *OsU2AF65B* under drought stress and its co-expression network, we revealed its potential role as a regulatory hub in plant drought response. The results indicate that *OsU2AF65B* is strongly associated with 13 genes across diverse functional categories, including transposon proteins, peptide transporters, immune-related proteins, and metabolic enzymes. This genetic diversity likely reflects the complex adaptive strategies for maintaining genome stability, redistributing nutrients, and remodeling metabolism under drought stress. Notably, the co-expression of transposon-related genes suggests that *OsU2AF65B* may maintain genomic stability by regulating transposon activity, although its exact role in abiotic stress responses remains unclear [62]. In plants, transposon activity is tightly regulated, as abnormal activity can disrupt genomic stability. Under abiotic stresses, such as drought, maintaining genomic stability is crucial for plant adaptation and survival [63]. As a key splicing factor, U2AF65B may dynamically regulate isoform production by directly binding to the target gene’s pre-mRNA splicing sites or indirectly affecting the splicing efficiency of downstream genes via secondary splicing factors [62]. This splicing regulation may be crucial for drought responses, as it can precisely allocate the expression of different functional modules, optimizing adaptive responses. For example, the multicopper oxidase encoded by *LOC_Os01g04200*, which interacted with *OsU2AF65B* among the co-expression network, may contribute to lignin synthesis and ROS scavenging via membrane-bound/secreted isoforms [62]. In summary, *OsU2AF65B* may play a crucial regulatory role in plant drought response. It co-expresses with genes from multiple functional categories and may fine-tune adaptive responses via splicing regulation.

## 4. Materials and Methods

### 4.1. Sequence Identification of Plant U2AF65B Genes

To identify genes in the U2AF65B family, a protein BLAST search was performed on Phytozome v12.1.6 (https://phytozome.jgi.doe.gov/) (accessed on 21 September 2022) using the *Arabidopsis thaliana* U2AF65B protein (AT1G60900) sequence as a query [64]. This search targeted five categories of plant genome sequences. We identified 103 putative U2AF65B sequences from 36 plant species and obtained their locations, lengths, and functional descriptions for subsequent analysis.

### 4.2. Phylogenetic Analysis of U2AF65B Gene Families

A phylogenetic tree was constructed from the 103 protein sequences identified to elucidate the evolutionary relationships and clustering patterns within the U2AF65B gene family. Protein sequences were aligned using Muscle v3.8 [65], and phylogenetic relationships were inferred using the Bayesian method (Jones model) in MrBayes v3.2.2 [66]. The Bayesian analysis was set as follows: generations: 2,000,011; sampling frequency: 1000; number of runs: 2; number of chains: 4; burn-in fraction: 0.25; and burn-in: 5000. The resulting phylogenetic tree was visualized and edited using FigTree v1.4.3.38 (http://tree.bio.ed.ac.uk/software/Figtree) (accessed on 23 September 2022) and iTOL v5.7 (https://itol.embl.de) (accessed on 23 September 2022) [67,68].

### 4.3. Gene Structure, Protein Domain, and Motif Analysis

Gene structure information was retrieved from Phytozome v12.1.6, and protein domains were identified from NCBI input protein sequences (https://www.ncbi.nlm.nih.gov/) (accessed on 23 September 2022). The first 10 motifs were identified using MEME Suite v5.3.3 (http://meme-suite.org/tools/meme) (accessed on 23 September 2022) with the maximum expected value algorithm. This analysis used cDNA and protein sequences obtained from Phytozome v12.1.6. Higher scores indicated stronger motifs with better matches for the overall pattern [69]. Finally, the gene structure was visualized using TBtools v2.003 and iTOL v5.7.

### 4.4. Homology Modeling and Amino Acid Conservation Estimation

Homology modeling of U2AF65B was generated using templates from *A. thaliana* (Q8L716.1.A, 100% similarity), *O. sativa* (Q2QZL4.1.A, 100% similarity), *P. patens* (4tu7.1.B, 74.78% similarity), *C. reinhardtii* (A0A835W1N8.1.A, 96.44% similarity), and *Selaginella moellendorffii* (D8T825.1.A, 99.52% similarity) via the SWISS-MODEL website (https://swissmodel.expasy.org/) (accessed on 24 September 2022) [70,71]. Multiple sequence alignment of the *U2AF65B* genes was performed using NCBI with the default parameters. Among the alignment results, darker red shades highlight significant differences between the amino acid residues of gene members.

### 4.5. Cis-Acting Element Prediction

Subsequently, 1.5 kb promoter sequences of the U2AF65B gene family were retrieved from Phytozome and used as input in PlantCARE (http://bioinformatics.psb.ugent.be/webtools/plantcare/html/) (accessed on 24 September 2022) to predict *cis*-acting elements. The predicted elements were visualized using TBtools.

### 4.6. Plant Materials, Stress Treatments, RNA Extraction, and RT-qPCR Analysis

To assess the expression levels of *U2AF65B* genes under different stresses, we selected the model plant rice (*Oryza sativa* cv. Nipponbare) and analyzed the expression of U2AF65B in shoots and roots. The seedlings were hydroponically cultivated in a growth chamber at 25 °C/20 °C, with a 16 h/8 h (day/night) photoperiod and light intensity of 100 μmol m^−2^ s^−1^. Rice seeds were sterilized with 0.1% carbendazim solution for 24 h, rinsed, and soaked again in sterile water for another 24 h before hydroponic culturing on a seedling tray. On day 11, weak seedlings were removed, and Kimura B nutrient solution (Kulai Bo Technology Co., Ltd, Beijing, China.) was applied. At the two-true-leaf stage (culturing for approximately 22 days), seedlings were subjected to four stress treatments: low temperature (8 °C), drought (20% PEG6000), salt (100 mmol L^−1^ s NaCl), and cadmium (100 μmol L^−1^ CdSO_4_) [32]. *Arabidopsis* wild-type and *AtU2AF65B* mutant seeds were grown in 1/2 MS medium with or without 1 μmol L^−1^ ABA. They were cultured at 23 °C/20 °C, with a 16 h/8 h (day/night) photoperiod. Germination rates (days 3–7) and root lengths (day 7) were recorded. Photographs were taken, and ABA-treated *A. thaliana* seed germination was measured on day 13.

The shoots and roots of rice seedlings were sampled at 0, 3, 6, and 12 h post-treatment, and leaves of 4-week-old *Arabidopsis* plants (wild-type Col-0 and T-DNA insertion mutant *65b-2*) were sampled. Total RNA was extracted using TRIzol reagent (Invitrogen, Carlsbad, CA, USA). First-strand cDNA synthesis was performed using the Transcriptor First Strand cDNA Synthesis Kit (Roche, Basel, Switzerland). RT-qPCR was conducted using Fast Start Universal SYBR Green Master Mix (Roche, Basel, Switzerland) and a 7500 Real-time PCR System (Bio-Rad, Hercules, CA, USA). *OsACTIN-1* (*LOC_Os05g0438800*) and *AtACTIN* (*AT3G18780*) served as controls for normalization between samples in rice and *Arabidopsis*, respectively. The expression analysis and calculation methods were conducted according to Cao et al. [72]. The expression data for *U2AF65B* in *A. thaliana* were obtained from the eFP browser (http://bar.utoronto.ca/) (accessed on 24 September 2022), log-transformed (base = 2), and used to generate heat maps with TBtools v1.0 [73].

### 4.7. Subcellular Localization Analysis of U2AF65B Genes

The GenScript Protein Subcellular Localization Prediction Tool (PSORT II) (https://www.genscript.com/psort.html) (accessed on 23 September 2022) was employed to predict the expression sites of U2AF65B proteins in representative plant species. The results were then visualized using TBtools. To validate the accuracy of the bioinformatic prediction results, one *U2AF65B* gene in rice (*OsU2AF65B*) was selected, and its expression location was investigated using experimental methods. The full-length CDS of *OsU2AF65B* was amplified using gene-specific primers (Table S2) and cloned into the PGREENII vector via the ClonExpress II One Step Cloning Kit (Vazyme, Nanjing, China). Rice protoplasts were extracted from one-week-old Nipponbare seedling stems. Equimolar amounts of the constructed plasmids were mixed, and the protoplasts were transfected by adding the plasmid mixture using a 40% (*v*/*v*) PEG4000 solution in a 1:5 volume ratio. The mixture was incubated at 28 °C overnight and observed via laser confocal microscopy [32].

### 4.8. Transcriptomic Data Analysis and Co-Expression Network Construction

Transcriptomic data from *Nipponbare* shoot samples under drought conditions were retrieved from the Rice RNA-seq Database (https://plantrnadb.com/ricerna/) (accessed on 27 February 2025), including the treatment (Library ID: SRX1500163, SRX1500164, and SRX1500165) and control (Library ID: SRX1500160, SRX1500161, and SRX1500162) groups. Differential expression analysis was performed using DESeq2, with screening criteria set as |log_2_FoldChange| > 1 and *p*-value < 0.05. Based on the DEGs, a co-expression network was constructed, and co-expression modules (communities) were identified via the online BioDeep One-Stop Research Service Big Data Cloud Platform (https://www.biodeep.cn/home) (accessed on 27 February 2025).

## 5. Conclusions

We identified 103 *U2AF65B* genes across 36 plant species and conducted comprehensive bioinformatics analyses of this gene family. The results indicated that *U2AF65B* genes were relatively conserved and responded to various external stimuli. The OsU2AF65B protein was found to be localized in the nucleus and the cytoplasm. In the *AtU2AF65B* mutant, root length significantly increased, resulting in greater seedling biomass. Under ABA induction, the germination rate of *AtU2AF65B* mutant seeds significantly increased. However, further research is needed to elucidate the molecular mechanisms regulating splicing to control plant growth and stress responses.

## Data Availability

All data generated in this study are included in this article and its supplementary files. The plant genome sequence information was obtained from Phytozome v12.1.6 (https://phytozome.jgi.doe.gov/) (accessed on 21 September 2022)(Table S1), promoter structure prediction information was obtained from PlantCARE (http://bioinformatics.psb.ugent.be/webtools/plantcare/html/) (accessed on 24 September 2022), subcellular localization prediction was obtained from the GenScript website (https://www.genscript.com/psort.html) (accessed on 23 September 2022) (Figure S6), homology modeling was performed using the SSWISS-MODEL website (https://swissmodel.expasy.org/) (accessed on 24 September 2022), gene and protein conserved motifs were obtained from MEME (http://meme-suite.org/tools/meme) (accessed on 24 September 2022) (Figure S2), phosphorylation site prediction was obtained from the NetPhos-3.1 website (http://www.cbs.dtu.dk/services/NetPhos/) (accessed on 24 September 2022) (Figure S4), and amino acid sequence frequency differences were assessed via multiple sequence alignment using the NCBI Multiple Sequence Alignment Viewer from NCBI (https://www.ncbi.nlm.nih.gov/) (accessed on 23 September 2022) (Figure S3). The data used and analyzed in this study can be obtained from the above-mentioned websites.

## Supplementary Materials

The following supporting information can be downloaded at https://www.mdpi.com/article/10.3390/ijms26083901/s1.

## Abbreviations

AS	Alternative splicing
ABA	Abscisic acid
pre-mRNA	Precursor messenger RNA
RRM	RNA recognition motif
ERE	Estrogen response element
MeJA	Methyl jasmonate
ABRE	ABA-responsive elements
ARE	Antioxidant Response Element
DEG	Differentially expressed genes
