# Supplementary material for: A Comprehensive Analysis of the Alternative Splicing Co-Factor U2AF65B Gene Family Reveals Its Role in Stress Responses and Root Development
Source: Int J Mol Sci. 2025 Apr 20;26(8):3901. doi: 10.3390/ijms26083901 (PMC12027700; doi:10.3390/ijms26083901)
Supplement: Supplementary file 1 [file ijms-26-03901-s001.zip › Supplymentary Figures.pdf]

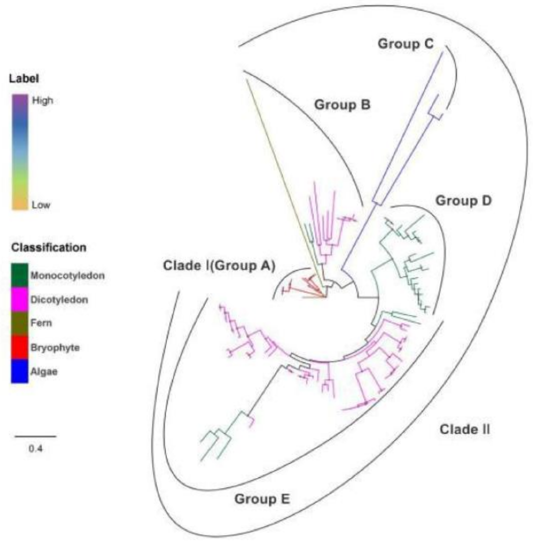

Figure S1. Evolutionary tree of 103 U2AF65B proteins. Phylogenetic analysis of the plant *U2AF65B* gene family was beautified using iTOL software.

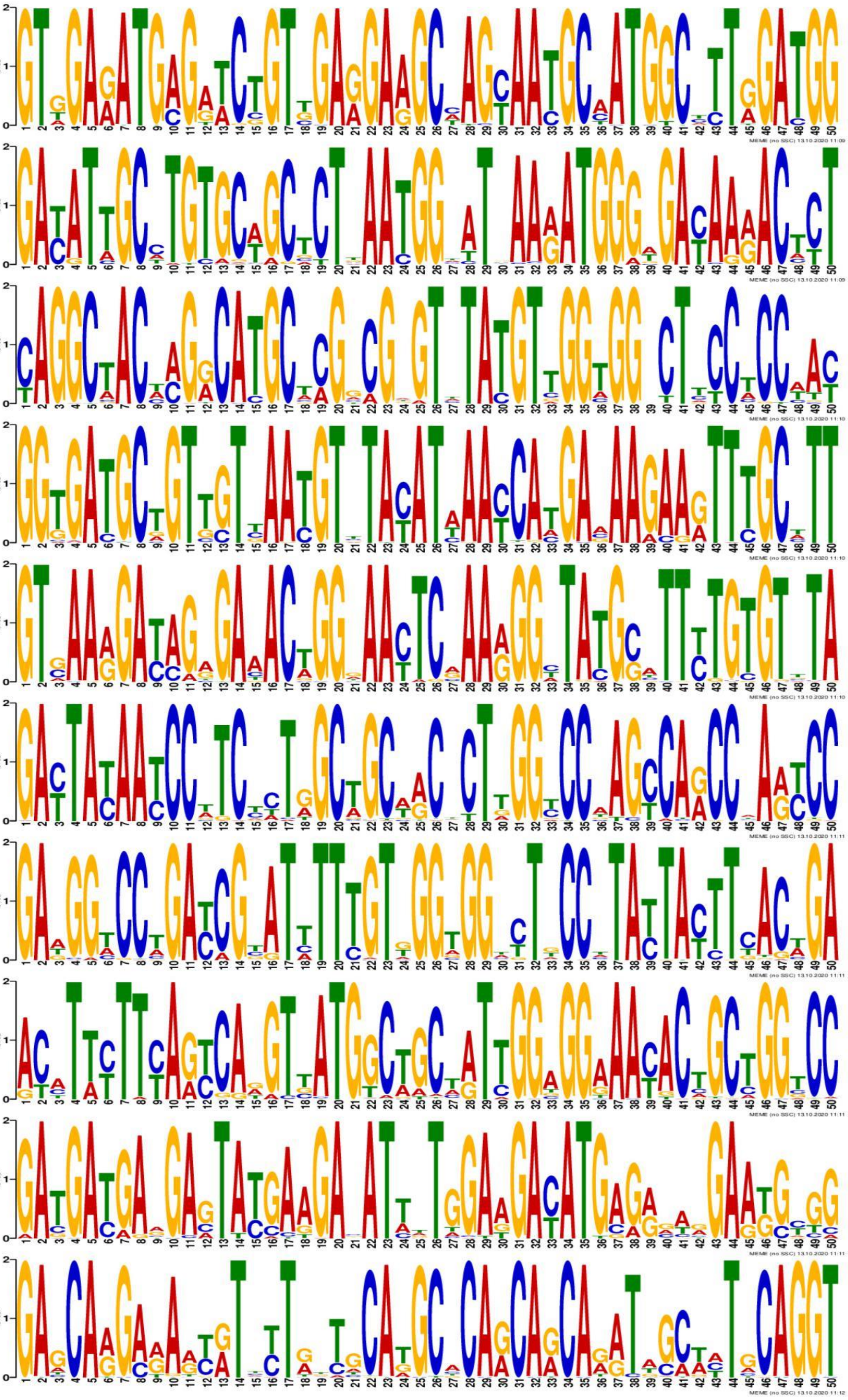

Figure S2. Motif analysis of U2AF65B proteins. Consensus sequences of the top ten identified motifs are listed.

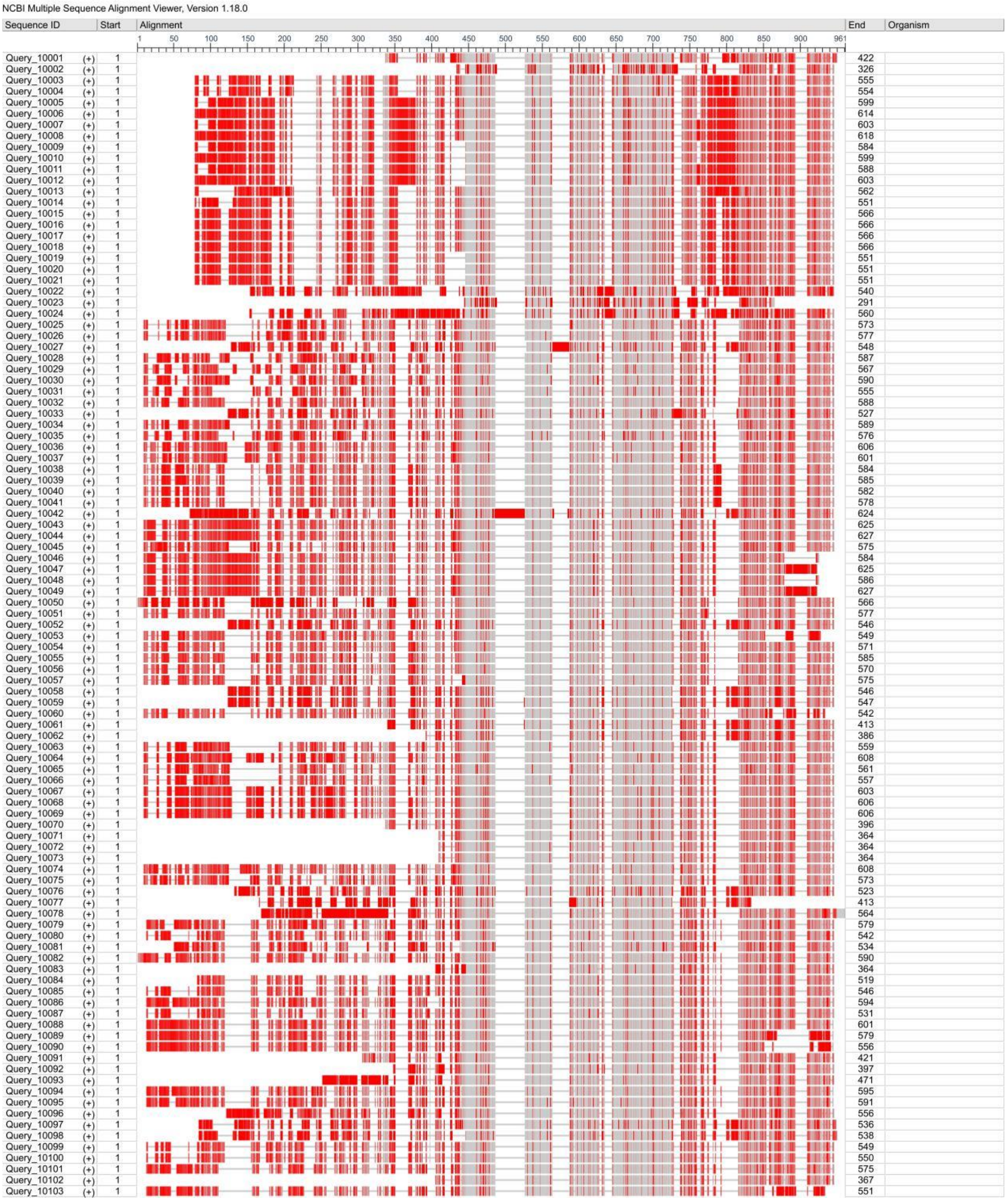

Figure S3. The multiple sequence alignment of RRM domains for conservative analysis. Multiple sequence alignment of RRM domain based on Frequency-Based Difference. Darker shades of red indicate greater differences from residues in other rows at that position. The sequences are arranged from

top to bottom according to the phylogenetic tree.

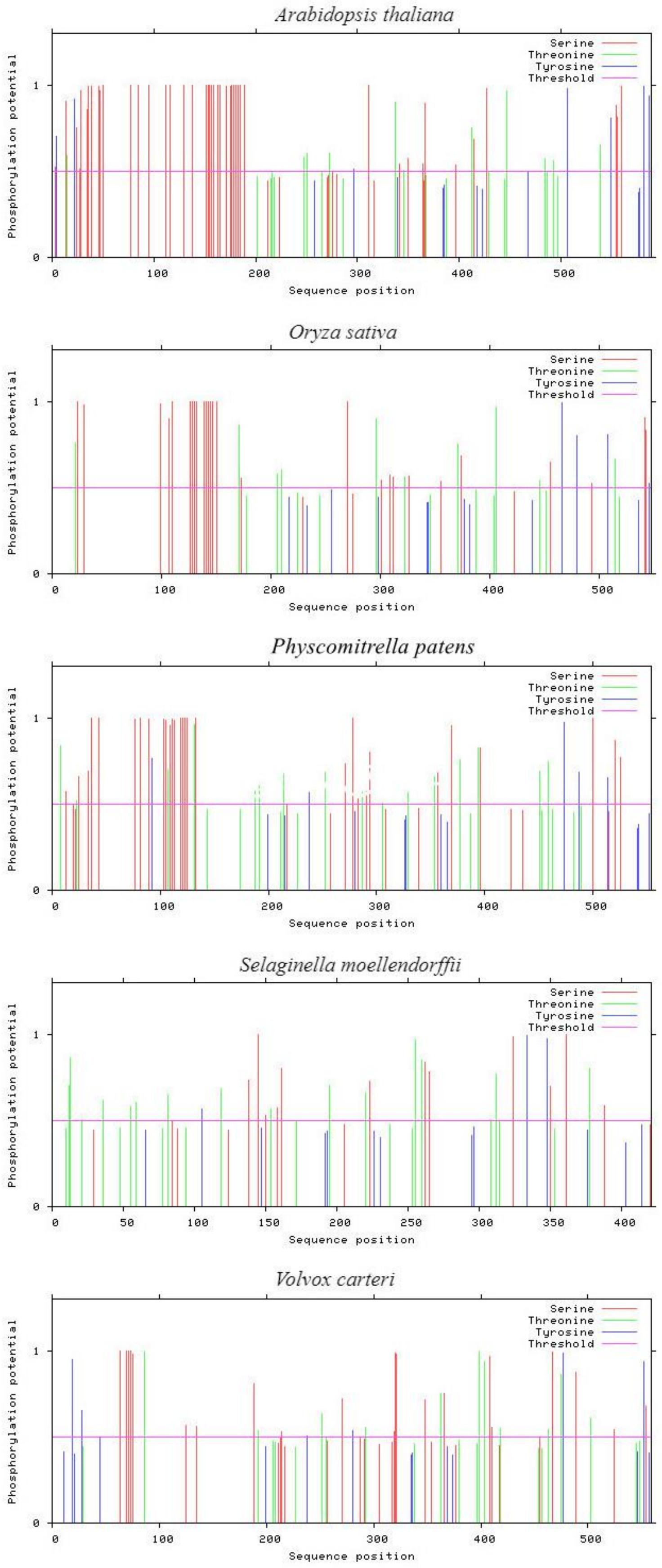

Figure S4. Phosphorylation site prediction map.

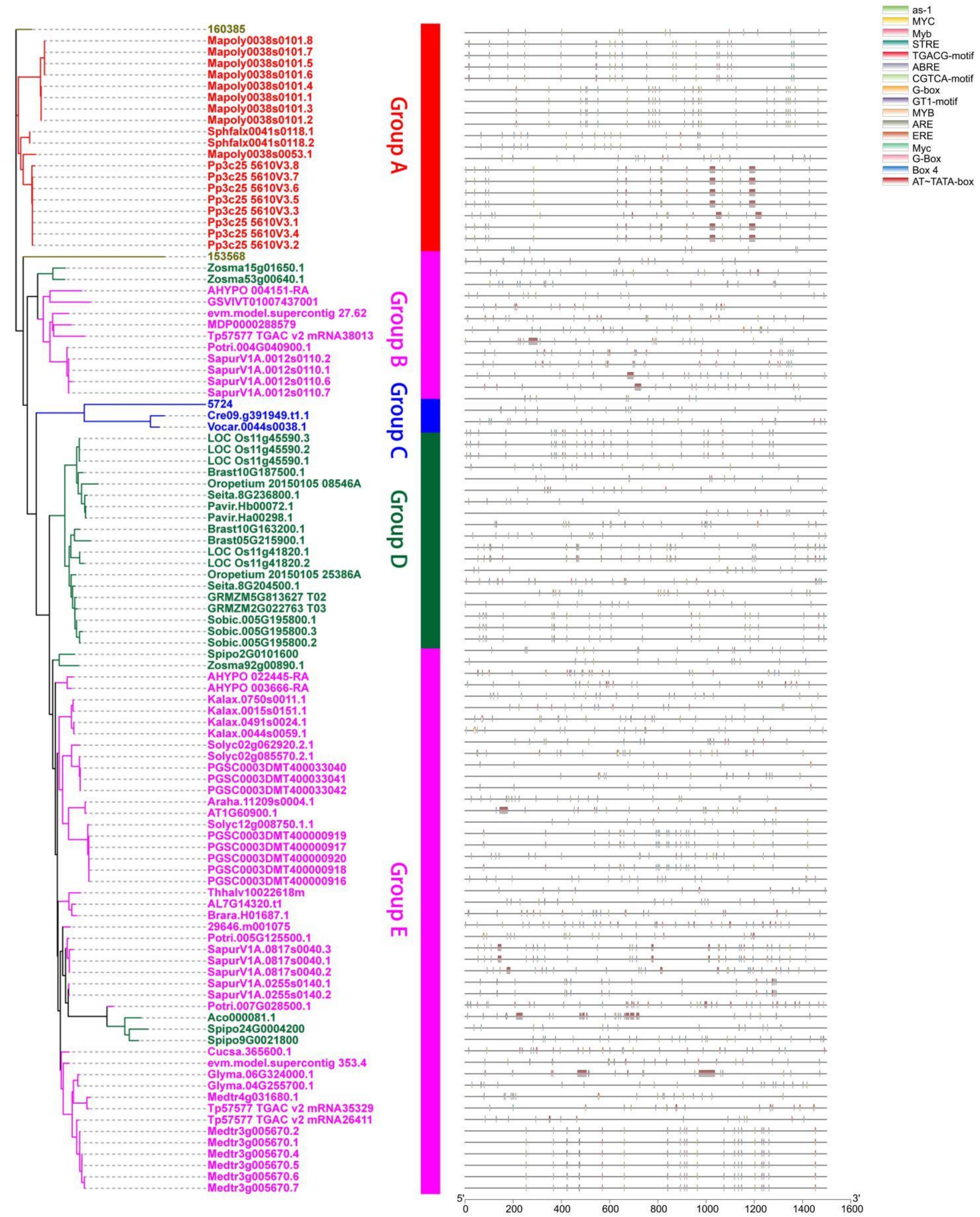

Figure S5. ***Cis*-acting elements analysis in the promoters of plant U2AF65B genes.** *Cis*-acting elements are represented by rectangles with various colors. These *cis*-acting elements are labeled along the upstream promoter sequences of 1.5-kb promoter region isolated from each *U2AF65B* gene based on their relative nucleotide positions to the transcript start site.

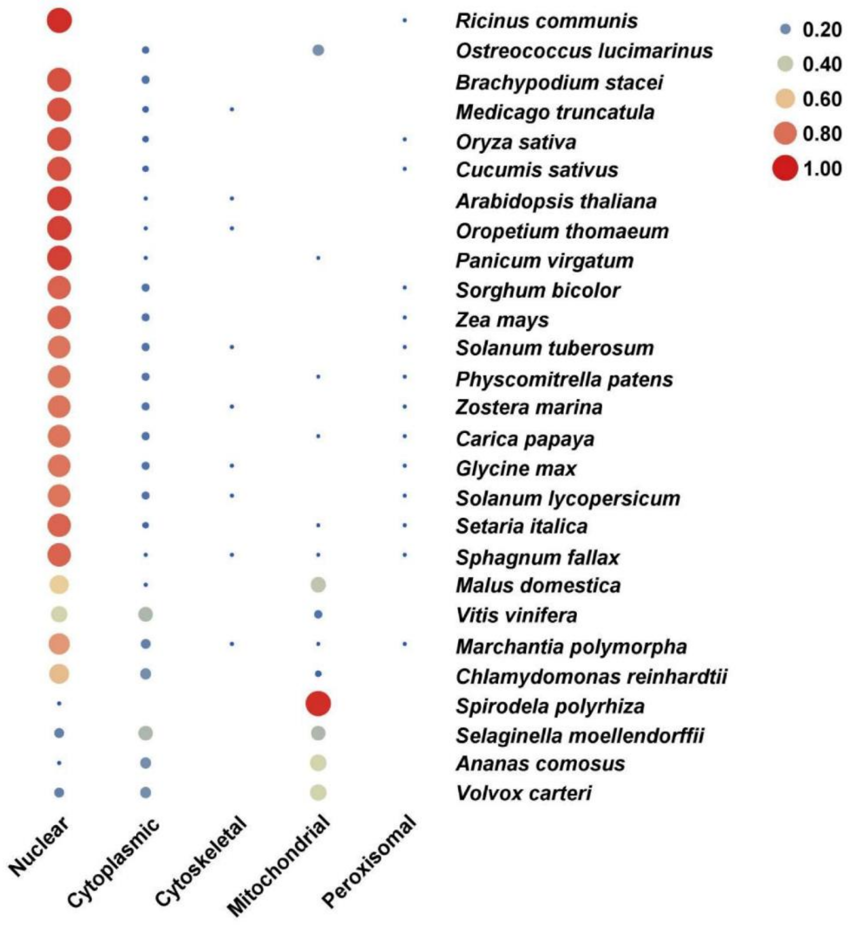

Figure S6. Subcellular localization prediction analysis of selected U2AF65B proteins. Bioinformatics prediction of subcellular localization based on their amino acid sequences to determine the probability of biomacromolecules within the cell.

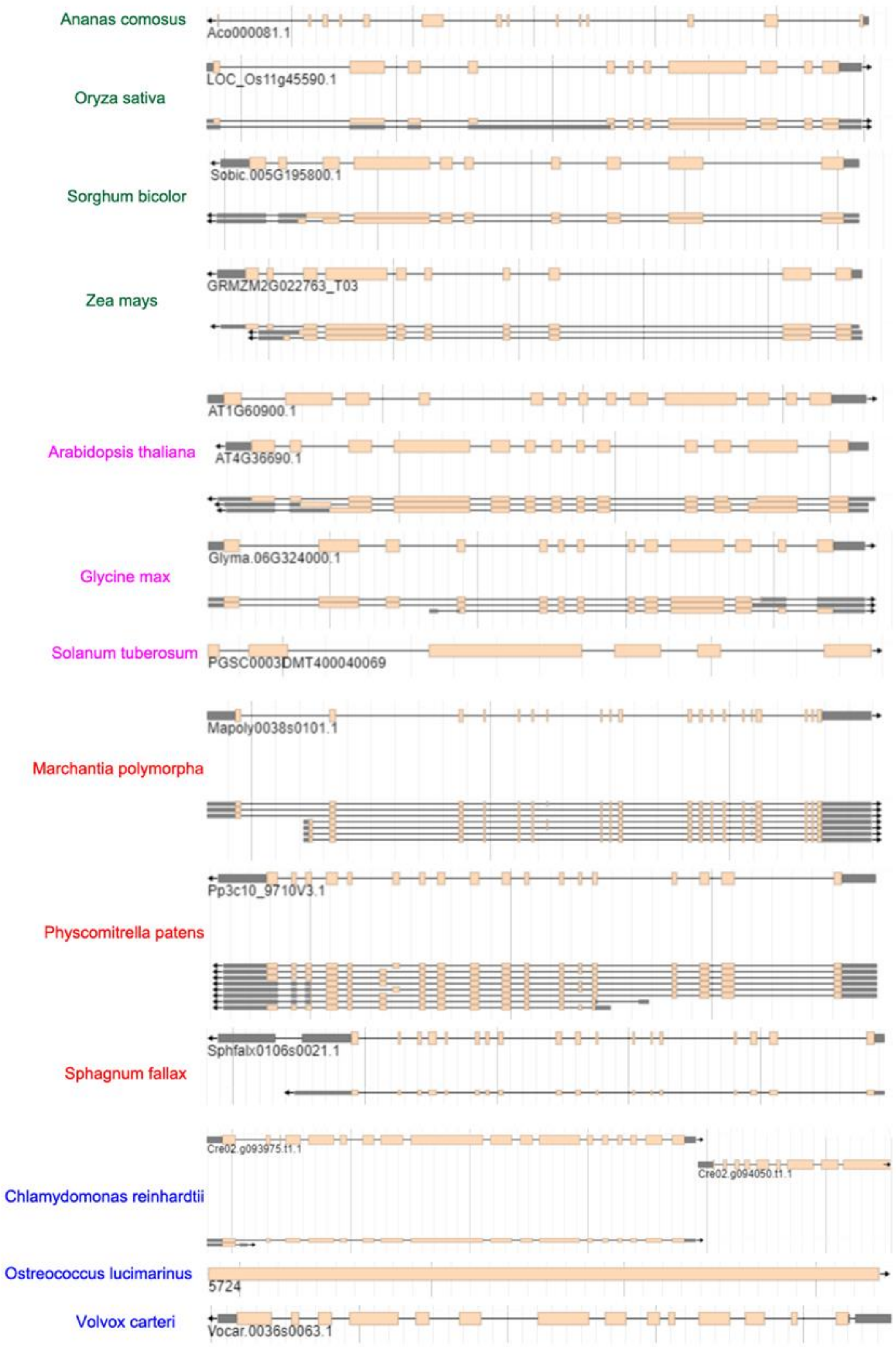

Figure S7. Alternative splicing (AS) profile of U2AF65B genes. Summary of annotated AS isoforms. Colors: green (monocotyledons), pink (dicotyledons), red (bryophytes), blue (algae).

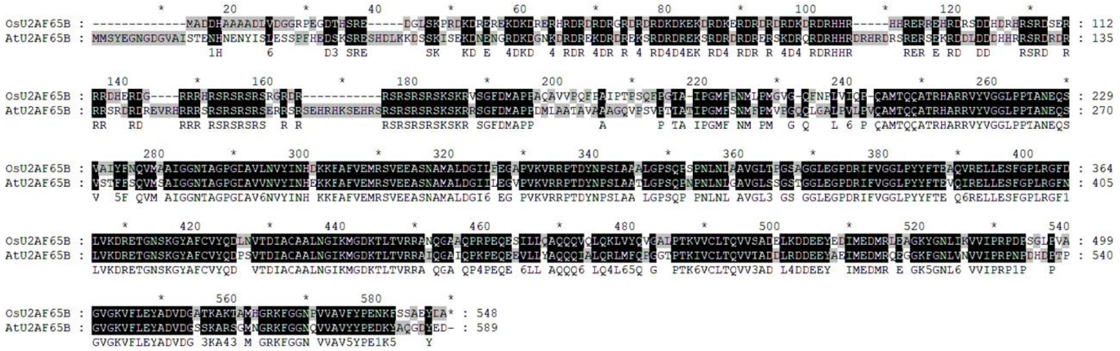

Figure S8. Amino acid sequence alignment of U2AF65B in rice and *Arabidopsis*.

Black background indicates identical amino acids, gray or white background represents different amino acids, and the bottom row shows the consensus amino acids shared between the two species.

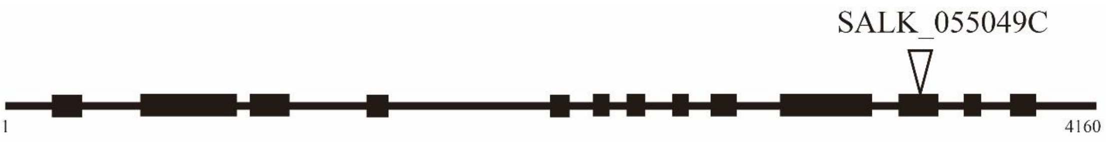

Mutant	Gene	Locus	Germplasm Name	Site
65b-2	U2AF65B	AT1G60900	SALK_055049C	exon

Figure S9. Information on mutant 65b-2 obtained from TAIR.
