# Supplementary material for: A Comprehensive Analysis of the Alternative Splicing Co-Factor U2AF65B Gene Family Reveals Its Role in Stress Responses and Root Development
Source: Int J Mol Sci. 2025 Apr 20;26(8):3901. doi: 10.3390/ijms26083901 (PMC12027700; doi:10.3390/ijms26083901)
Supplement: Supplementary file 1 [file ijms-26-03901-s001.zip › Supplymentary Tables.pdf]

Table S1. Summary of 103 U2AF65B genes across 36 plant species.

Subfamily	Groups	Organism	Transcript ID
Blue	Algae	*Chlamydomonas reinhardtii*	Cre09.g391949.t1.1
		Ostreococcus lucimarinus	5724
		Volvox carteri	Vocar.0044s0038.1
Red	Bryophytes	*Marchantia polymorpha*	Mapoly0038s0101.8
			Mapoly0038s0101.3
			Mapoly0038s0101.7
			Mapoly0038s0101.1
			Mapoly0038s0101.5
			Mapoly0038s0101.2
			Mapoly0038s0101.6
			Mapoly0038s0101.4
			Mapoly0038s0053.1
		Physcomitrella patens	Pp3c25_5610V3.8
			Pp3c25_5610V3.4
			Pp3c25_5610V3.3
			Pp3c25_5610V3.1
			Pp3c25_5610V3.2
			Pp3c25_5610V3.6
			Pp3c25_5610V3.7
			Pp3c25_5610V3.5
		Sphagnum fallax	Sphfalx0041s0118.1
			Sphfalx0041s0118.2
Pink	Dicotyledon	*Amaranthus hypochondriacus*	AHYPO_022445-RA
			AHYPO_003666-RA
			AHYPO_004151-RA
		Arabidopsis halleri	Araha.11209s0004.1
		Arabidopsis lyrata	AL7G14320.t1
		Arabidopsis thaliana	AT1G60900.1
		Brassica rapa	Brara.H01687.1
		Carica papaya	evm.model.supercontig_353.4
			evm.model.supercontig_27.62
		Cucumis sativus	Cucsa.365600.1
		Eutrema salsugineum	Thhalv10022618m
		Glycine max	Glyma.06G324000.1
			Glyma.04G255700.1
		Kalanchoe laxiflora	Kalax.0750s0011.1
			Kalax.0015s0151.1

			**Kalax.0491s0024.1**
**Pink**	**Dicotyledon**	*Kalanchoe laxiflora*	**Kalax.0044s0059.1**
		Malus domestica	**MDP0000288579**
		Medicago truncatula	**Medtr3g005670.2**
			**Medtr3g005670.1**
			**Medtr4g031680.1**
			**Medtr3g005670.4**
			**Medtr3g005670.6**
			**Medtr3g005670.5**
			**Medtr3g005670.7**
		Populus trichocarpa	**Potri.007G028500.1**
			**Potri.005G125500.1**
			**Potri.004G040900.1**
		Ricinus communis	**29646.m001075**
		Salix purpurea	**SapurV1A.0817s0040.1**
			**SapurV1A.0255s0140.1**
			**SapurV1A.0817s0040.2**
			**SapurV1A.0255s0140.2**
			**SapurV1A.0012s0110.2**
			**SapurV1A.0012s0110.1**
			**SapurV1A.0817s0040.3**
			**SapurV1A.0012s0110.6**
			**SapurV1A.0012s0110.7**
		Solanum lycopersicum	**Solyc02g085570.2.1**
			**Solyc12g008750.1.1**
			**Solyc02g062920.2.1**
		Solanum tuberosum	**PGSC0003DMT400033041**
			**PGSC0003DMT400000918**
			**PGSC0003DMT400000919**
			**PGSC0003DMT400000917**
			**PGSC0003DMT400000916**
			**PGSC0003DMT400000920**
			**PGSC0003DMT400033042**
			**PGSC0003DMT400033040**
		Trifolium pratense	**Tp57577_TGAC_v2_mRNA26411**
			**Tp57577_TGAC_v2_mRNA35329**
			**Tp57577_TGAC_v2_mRNA38013**
		Vitis vinifera	**GSVIVT01007437001**

**Olive Green**	**Pteridophyta**	*Selaginella moellendorffii*	**160385**
			**153568**
**Green**	**Monocotyledon**	*Ananas comosus*	**Aco000081.1**
**Green**	**Monocotyledon**	*Brachypodium stacei*	**Brast10G163200.1**
			**Brast10G187500.1**
			**Brast05G215900.1**
		Oryza sativa	**LOC_Os11g45590.2**
			**LOC_Os11g45590.1**
			**LOC_Os11g41820.1**
			**LOC_Os11g45590.3**
			**LOC_Os11g41820.2**
		Oropetium thomaeum	**Oropetium_20150105_25386A**
			**Oropetium_20150105_08546A**
		Panicum virgatum	**Pavir.Hb00072.1**
			**Pavir.Ha00298.1**
		Setaria italica	**Seita.8G204500.1**
			**Seita.8G236800.1**
		Sorghum bicolor	**Sobic.005G195800.1**
			**Sobic.005G195800.3**
			**Sobic.005G195800.2**
		Spirodela polyrhiza	**Spipo2G0101600**
			**Spipo24G0004200**
			**Spipo9G0021800**
		Zea mays	**GRMZM5G813627_T02**
			**GRMZM2G022763_T03**
		Zostera marina	**Zosma92g00890.1**
			**Zosma15g01650.1**
			**Zosma53g00640.1**

Table S2. Primer information used in this study.

Gene	Sequence（5′-3′）
OsU2AF65B	F: CCTGTTGTTTGGTGTTACTTAAGCTTATGGCGGACGACCACGC
	R: TCCTCGCCCTTGCTCACCATGGATCCCTACGCGTCATATTCCGCACTAGA
OsACTIN-1	F: CTTCATAGGAATGGAAGCTGCGGGTA
	R: CGACCACCTTGATCTTCATGCTGCTA
AtACTIN	F: GCCCAGAAGTCTTGTTCCAG
	R: TTGGAGATCCACATCTGCTG
OsU2AF65B RT-QPCR	F: ACAAAGGTTGTGTGCCTGAC
	R: TGCCTGCTTCCAATCTCATG
